# Identification of *Beilschmiedia tsangii* Root Extract as a Liver Cancer Cell–Normal Keratinocyte Dual-Selective NRF2 Regulator

**DOI:** 10.3390/antiox10040544

**Published:** 2021-04-01

**Authors:** Yi-Siao Chen, Hsun-Shuo Chang, Hui-Hua Hsiao, Yih-Fung Chen, Yi-Ping Kuo, Feng-Lin Yen, Chia-Hung Yen

**Affiliations:** 1 Ph.D. Program in Environmental and Occupational Medicine, College of Medicine, Kaohsiung Medical University, Kaohsiung 80708, Taiwan; wl01420323@gmail.com; 2 Institute of Molecular and Genomic Medicine, National Health Research Institute, Miaoli County 35053, Taiwan; 3 Graduate Institute of Natural Products, College of Pharmacy, Kaohsiung Medical University, Kaohsiung 80708, Taiwan; hschang@cc.kmu.edu.tw (H.-S.C.); yihfungchen@kmu.edu.tw (Y.-F.C.); kone5207@gmail.com (Y.-P.K.); 4 National Natural Product Libraries and High-Throughput Screening Core Facility, Kaohsiung Medical University, Kaohsiung 80708, Taiwan; 5 School of Pharmacy, College of Pharmacy, Kaohsiung Medical University, Kaohsiung 80708, Taiwan; 6 Drug Development and Value Creation Research Center, Kaohsiung Medical University, Kaohsiung 80708, Taiwan; kmckmc19@gmail.com; 7 Division of Hematology and Oncology, Department of Internal Medicine, Kaohsiung Medical University Hospital, Kaohsiung 80708, Taiwan; huhuhs@cc.kmu.edu.tw; 8 Center for Liquid Biopsy, Kaohsiung Medical University, Kaohsiung 80708, Taiwan; 9 Faculty of Medicine, Kaohsiung Medical University, Kaohsiung 80708, Taiwan; 10 Department of Medical Research, Kaohsiung Medical University Hospital, Kaohsiung 80708, Taiwan; 11 Department of Fragrance and Cosmetic Science, College of Pharmacy, Kaohsiung Medical University, Kaohsiung 80708, Taiwan

**Keywords:** NRF2, high-throughput screen, adjuvant treatment, natural product

## Abstract

Transcription factor nuclear factor erythroid 2-related factor 2 (NRF2) plays a crucial role in regulating the expression of genes participating in cellular defense mechanisms against oxidative or xenobiotic insults. However, there is increasing evidence showing that hyperactivation of NRF2 is associated with chemoresistance in several cancers, including hepatocellular carcinoma (HCC), thus making NRF2 an attractive target for cancer therapy. Another important issue in cancer medication is the adverse effects of these substances on normal cells. Here, we attempted to identify a dual-selective NRF2 regulator that exerts opposite effects on NRF2-hyperactivated HCC cells and normal keratinocytes. An antioxidant response element driven luciferase reporter assay was established in Huh7 and HaCaT cells as high-throughput screening platforms. Screening of 3,000 crude extracts from the Taiwanese Indigenous Plant Extract Library resulted in the identification of *Beilschmiedia tsangii* (BT) root extract as a dual-selective NRF2 regulator. Multiple compounds were found to contribute to the dual-selective effects of BT extract on NRF2 signaling in two cell lines. BT extract reduced NRF2 protein level and target gene expression levels in Huh7 cells but increased them in HaCaT cells. Furthermore, notable combinatory cytotoxic effects of BT extract and sorafenib on Huh7 cells were observed. On the contrary, sorafenib-induced inflammatory reactions in HaCaT cells were reduced by BT extract. In conclusion, our results suggest that the combination of a selective NRF2 activator and inhibitor could be a practical strategy for fine-tuning NRF2 activity for better cancer treatment and that plant extracts or partially purified fractions could be a promising source for the discovery of dual-selective NRF2 regulators.

## 1. Introduction

Liver cancer occupies the sixth place regarding cancer incidence and the fourth in cancer mortality rate worldwide (data source: http://gco.iarc.fr/today/home, accessed date 22 November 2020). Hepatocellular carcinoma (HCC), the major subtype of primary liver cancer, accounts for ~80% of cases [1]. Despite great advances in HCC management, the incidence and mortality rate of HCC are still high due to frequent tumor recurrence after treatment. Even sorafenib, a multiple kinase inhibitor and the first approved systemic therapy for advanced HCC, provides limited survival benefits. The failure of cancer therapy has been attributed to the existence of primary and acquired drug resistance mechanisms [2]. 

The nuclear factor erythroid-derived 2-like 2 (NRF2)-Kelch-like ECH-associated protein 1 (KEAP1) pathway is a redox- and xenobiotic-sensitive signaling axis that functions to protect cells against oxidative stress and environmental toxicants through the induction of cytoprotective genes. NRF2 participates in the basal activity and coordinates the induction of genes encoding numerous antioxidant and detoxifying enzymes and related proteins such as catalase (CAT), superoxide dismutase (SOD), NAD(P)H:quinone oxidoreductase-1 (NQO1), heme oxygenase-1 (HO1), glutamate cysteine ligase (GCLC), glutathione *S*-transferase (GST), glutathione peroxidase (GPX), and thioredoxin (TXN) [3,4]. NRF2 is held in the cytoplasm as an inactive complex bound to KEAP1, which facilitates the ubiquitination of NRF2 [5]. Modification of KEAP1 cysteine residues results in inhibition of NRF2 ubiquitylation and stabilization of NRF2. Accumulated NRF2 then translocates into the nucleus where it binds to a small MAF protein and activates transcription of genes containing antioxidant response elements (AREs) in their regulatory regions [3]. Due to its cytoprotective function, NRF2 has been traditionally considered as a tumor suppressor and target for chemoprevention [6]. However, recent genetic analyses of human cancer revealed that NRF2 could be oncogenic and cause resistance to chemotherapy. Moreover, abnormal high expression of NRF2 is frequently seen in human tumor specimens and is correlated with poor prognosis [7,8]. In addition, evidence from studies in cancer cell lines and in experimental animals supported the capability of NRF2 to enhance drug resistance against a diverse range of drugs, such as cisplatin, carboplatin, 5‑fluorouracil, paclitaxel, bleomycin, doxorubicin, and etoposide [9,10]. Thus, NRF2 is also regarded as an important pharmacological target for anticancer therapy. 

In agreement with this notion, natural products, natural product derivatives, and small molecules that suppress NRF2 activity have been shown to render cancer cells more sensitive to anticancer therapies [11,12,13]. Singh and colleagues used KEAP1-mutated non-small-cell lung cancer (NSCLC) cell lines stably carrying ARE luciferase reporter gene fragment as screening platforms and identified ML385 as a potent NRF2 inhibitor that directly binds to NRF2 then interrupts the binding of NRF2–MAFG complex to ARE. ML385 shows specific and selective cytotoxicity and potentiates the toxicity of chemotherapy drugs in KEAP1-mutant NSCLC cells [14]. However, the effects of NRF2 inhibitors on normal cells, which are vulnerable to anticancer agents, should also be considered. For instance, cisplatin and doxorubicin, widely used anticancer agents, are well known for causing nephrotoxicity and cardiotoxicity, respectively, which limits their clinical use [15,16]. Similarly, sorafenib, a targeted therapy agent, can cause cutaneous side effects. Although the skin toxicity of sorafenib is not a life-threatening side effect, it significantly affects quality of life and leads to dose reductions [17]. Nonselective NRF2 inhibitors could worsen anticancer-agent-induced adverse effects. On the other hand, natural compound NRF2 activators such as sulforaphane and curcumin have been shown to prevent side effects caused by anticancer drugs [18]. Thus, more efforts are needed to search for better and more selective NRF2 regulators that can potentiate anticancer treatment and at the same time alleviate the collateral toxic effects in noncancerous tissues [18,19]. Therefore, in this study, we aimed to identify selective NRF2 regulators that could specifically suppress NRF2 function in liver cancer cells but enhance it in normal keratinocytes. A pair of high-throughput screening platforms was established and used for screening a natural product library that contains 3000 crude extracts prepared from 1336 Taiwanese indigenous plants [20]. The crude extract of root of *Beilschmiedia tsangii* Merr. (BT extract) was identified as a selective NRF2 regulator that inhibited NRF2 activity in Huh7 cells and activated it in HaCaT cells. Furthermore, the effects of BT extract on sorafenib-induced cytotoxicity in Huh7 cells and sorafenib-induced adverse effects in HaCaT cells were evaluated.

## 2. Materials and Methods

### 2.1. Taiwanese Indigenous Plant Extract Library (TIP Library) 

The details of the TIP library were described in a previous report [20]. 

### 2.2. Cell Cultures and Transfection

HEK293T and liver cell lines including Sk-hep1, HepG2, Hep3B, PLC/PRF/5, Huh7, and Mahlavu were cultured as described previously [21]. HaCaT cells were cultured in Dulbecco’s Modified Eagle’s Medium (DMEM) (Thermo Fisher Scientific, Taipei, Taiwan) with 10% heat-inactivated fetal bovine serum (Thermo Fisher Scientific), penicillin (100 U/mL), and streptomycin (100 μg/mL). Lentivirus-infected cells were grown in complete DMEM supplemented with either hygromycin along (100 μg/mL, Thermo Fisher Scientific) or puromycin (1 μg/mL) and hygromycin (100 μg/mL) together. TurboFect Reagent (Fermentas, Hanover, MD, USA) was used for plasmid DNA transfection according to the manufacturers’ instructions.

### 2.3. Quantitative Real-Time PCR (QPCR)

RNA was prepared by using TRIzol Reagent (Thermo Fisher Scientific) and was reverse transcribed into cDNA using a TOOLs Easy Fast RT Kit (TOOLs Biotechnology, New Taipei City, Taiwan). QPCR was performed on an ABI StepOne Plus System (Applied Biosystems, Foster City, CA, USA) using the KAPA SYBR FAST qPCR Master Mix (2X) Kit (KAPA Biosystems, Woburn, MA, USA). The mRNA level was normalized with the glyceraldehyde 3-phosphate dehydrogenase (GAPDH) mRNA level. The following primers were used: HO1 (NM_002133) forward: 5′-GCCAGCAACAAAGTGCAAG and reverse: 5′-GAGTGTAAGGACCCATCGGA; NQO1 (NM_000903) forward: 5′-TGCAGCGGCTTTGAAGAAGAAAGG and reverse: 5′-TCGGCAGGATACTGAAAGTTCGCA; GCLC (NM_001498) forward: 5′-CTGGGGAGTGATTTCTGCAT and reverse: 5′-AGGAGGGGGCTTAAATCTCA; ABCC2 (NM_000392) forward: 5′-TACCTAGGCACATGGCTCCT and reverse: 5′- AGAACAGGCAGGAGTAGGCT; G6PD (NM_000402) forward: 5′-CAACATCGCCTGCGTTA and reverse: 5′-CTTGACCTTCTCATCACGG; COX2 (NM_000963) forward: 5′-GTTCCACCCGCAGTACAGAA and reverse: 5′-AGGGCTTCAGCATAAAGCGT; IL-1β (NM_000576) forward: 5′-TGAGCTCGCCAGTGAAATGA and reverse: 5′-AGATTCGTAGCTGGATGCCG; TNF-α (NM_000594) forward: 5′- TGGCGTGGAGCTGAGAGATA and reverse: 5′-CTTGGTCTGGTAGGAGACGG; MMP1 (NM_002421) forward: 5′-GAGATCATCGGGACAACTCTCCT and reverse: 5′-GTTGGTCCACCTTTCATCTTCAT; MMP3 (NM_002422) forward: 5′-TGAAATTGGCCACTCCCTGG and reverse: 5′-GGAACCGAGTCAGGTCTGTG; GAPDH (NM_002046) forward: 5′-GCAAATTCCATGGCACCGTCA and reverse: 5′-TCCTGGAAGATGGTGATGGGA.

### 2.4. Immunoblot

Cells were washed with PBS and harvested with RIPA lysis buffer (50 mM Tris (pH 7.5), 150 mM NaCl, 1mM EDTA, 1% NP-40, 0.1% SDS) supplemented with protease and phosphatase inhibitors (1 mM PMSF, 10 μg/mL Leupeptin, 50 μg/mL TLCK, 50 μg/mL TPCK, 1 μg/mL Aprotinin, 1 mM NaF, 5 mM NaPPi, and 10 mM Na_3_VO_4_). The cell lysates were cleared via 15 min spins at 13,000 rpm, 4 °C. Protein concentrations were determined by Bio-Rad Protein Assay (Bio-Rad, Hercules, CA, USA). The following antibodies were used: anti-NRF2 (GTX103322, GeneTex, Irvine, CA, USA), anti-GAPDH (60004-1g, Proteintech, Rosemont, IL, USA), anti-KEAP1 (10503-2-AP, Proteintech, Rosemont, IL, USA), anti-lamin B1 (66095-1-Ig, Proteintech, Rosemont, IL, USA).

### 2.5. Plasmids and Constructs

A 4 Kb NRF2 reporter fragment was generated by digesting the pGL4.37[luc2P/ARE/Hygro] (Promega Corporation, Madison, WI, USA) with *Ssp*I and *Sal*I. A pLKO.1-shLuc plasmid (clone#TRCN0000072249) purchased from National RNAi Core Facility (Academia Sinica, Taiwan) was digested by *Cla*I and *Kpn*I to generate a 5.3 Kb fragment that contained all required elements for lentiviral transduction and was used as backbone. The Quick Blunting Kit (New England Biolabs, Beverly MA, USA) was used to convert the incompatible 5′ or 3′ overhangs in these two fragments to blunt-end DNA according to manufacturers’ instructions. The map of the ligation product of these two fragments was confirmed by restriction enzyme digestion with *Ssp*I and *Eco*RV. The resultant plasmid was then designated as pLV-ARE-Luc_R (Figure S1). Two plasmids encoding different shRNAs for NRF2, namely shNRF2-1 (5’AGTTTGGGAGGAGCTATTATC, clone#: TRCN0000007555) and shNRF2-2 (5’GCTCCTACTGTGATGTGAAAT, clone#: TRCN0000273494); the control plasmids for the RNA interference (pLKO.1-shSCR); the packaging plasmid (pCMV-DR8.91); and the envelope plasmid (pMD.G) were obtained from National RNAi Core Facility (Academia Sinica, Taiwan).

### 2.6. Viral Infection

HEK293T cells were used for pseudotyped lentivirus preparation as described previously [21]. To generate stable cell lines, cells were infected with pseudotyped lentivirus-containing medium with the presence of polybrene (8 μg/mL) for 24 h. Stable cells were then selected by antibiotic-containing medium 48 h after infection.

### 2.7. Luciferase Reporter Assay and Cell Viability Assay

Luciferase reporter assay and cell viability assay were performed based on methods described in a previous paper [22]. Relative luciferase activity (RLA) was calculated by normalizing luciferase activity to cell viability. The average RLA of DMSO wells was defined as the control and attributed a relative NRF2 activity of 100%. Combination index values were determined using the CompuSyn 1.0 software.

### 2.8. Subcellular Fractionation

Nuclear and cytoplasmic proteins were collected using EPIXTRACT Nuclear Protein Isolation Kit (ENZ-45016, Enzo Life Sciences, Ann Arbor, MI, USA) according to the manufacturer’s instructions.

### 2.9. Statistical Analyses

All data were analyzed using GraphPad Prism 6.01 software (La Jolla, CA, USA). Differences in multiple groups were compared with one-way analysis of variance (ANOVA) followed by Dunnett’s comparison test. In combination study, the data of cell viability assay were analyzed by two-way ANOVA. A *P*-value < 0.05 was considered statistically significant. The Z’-factor was used to quantify the suitability of the luciferase reporter assay for high-throughput screening (HTS) [23]. A platform with a Z’-factor > 0.5 represents an excellent assay for HTS. The Z’-factor was calculated based on the following equation: Z’ = 1 − (3 SD of positive controls + 3 SD of negative controls)/│mean of positive controls − mean of negative controls│. 

## 3. Results

### 3.1. Establishment of Drug Screening Platform and Identification of Beilschmiedia tsangii Merr. Extract as a Selective NRF2 Regulator 

Although persistent activation of NRF2 signaling has been reported in several cancer cell lines, particularly lung cancer, the profile of NRF2 activation among liver cancer cell lines remains largely unknown [8]. We thus analyzed the basal NRF2 protein level and the expression of NQO1, an NRF2 target gene, in a panel of five liver cancer cell lines, namely Sk-hep1, HepG2, Hep3B, PLC/PRF/5, Huh7, and Mahlavu. NRF2, as well as NQO1, is expressed at the highest abundance in Huh7 cells (Figure S2). These results suggest that Huh7 cells could be considered as an HCC cell line with persistent NRF2 activation, and they were used as one screening cell model in this study. On the other hand, to identify selective NRF2 regulators, we used normal keratinocyte cell line HaCaT cells as the other cell model, because hand–foot skin reaction (HFSR) is the most common adverse effect related to sorafenib treatment, which remains the only approved systemic therapy for advanced HCC [17]. The result confirmed a much lower NRF2 protein expression in HaCaT cells (Figure S2A).

**Figure 1:**
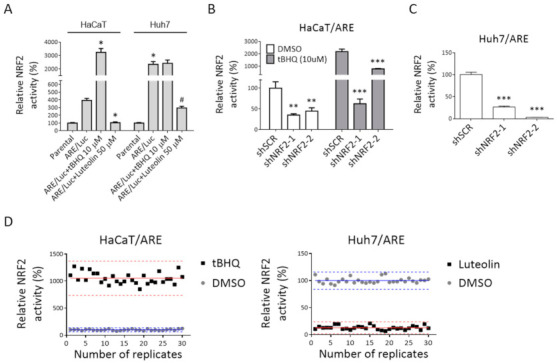
Establishment of high-throughput screening (HTS) platforms for selective NRF2 regulator screening. (**A**) NRF2 reporter fragment (ARE/Luc) was stably expressed in HaCaT cells (HaCaT/ARE) and Huh7 cells (Huh7/ARE). Luciferase activity and cell viability were measured in these two stable cell lines and their corresponding parental cells. Relative luciferase activity (RLA) was calculated as described in Section 2. Luteolin and tert-butylhydroquinone were used as NRF2 inhibitor and activator, respectively. The average of RLA in parental cells was used as a standard for 100%. The graph shows the means ± SD (*n* = 6). * indicates significant difference from HaCaT/ARE; # indicates significant difference from Huh7/ARE by one-way ANOVA. (**B**,**C**) Knock-down of NRF2 by shRNAs dramatically reduced NRF2 reporter activity in HaCaT/ARE cells (**B**) and Huh7/ARE cells (**C**). * indicates significant difference from control scramble shRNA (shSCR) group (** *p* < 0.01, *** *p* < 0.001, one-way ANOVA). (**D**) Variation test for platform evaluation. Huh7/ARE and HaCaT/ARE cells were determined in two experimental groups: DMSO (circles) and tBHQ (10 μM) or luteolin (50 μM) (squares). In each group, 30 wells were tested. The average of the DMSO group was used as a standard for 100%. Each dot represents NRF2 activity for one well. Solid lines represent the mean relative NRF2 activity, and dashed lines represent ± 3 standard deviations (SDs) for each group.

To monitor NRF2 activity in these two cell lines, a DNA fragment containing an antioxidant response element (ARE), a minimal promoter, a luciferase reporter gene Luc2P, a hygromycin resistant gene, and some other required elements was cloned into pLKO.1 lentiviral vector (Figure S1). The reporter fragment was then delivered into Huh7 and HaCaT cells by the lentiviral system. Cells stably carrying the NRF2 reporter fragment were selected and maintained by hygromycin-containing medium and were designated as Huh7/ARE and HaCaT/ARE cells. The existence of the reporter fragment in both cell lines was evidenced by remarkably elevated luciferase activities (Figure 1A). In addition, depletion of NRF2 in these two reporter cells (Figure S3) significantly reduced luciferase activities (Figure 1B,C), which confirm the specificity of these reporter platforms. It was also interesting to note that a significantly higher NRF2 reporter activity was found in Huh7/ARE cells compared to HaCaT/ARE cells, which was consistent with the higher expression level of NRF2 in Huh7 cells. Furthermore, tBHQ, an NRF2 activator, can further induce NRF2 activity in HaCaT/ARE cells, but not in Huh7/ARE cells. Luteolin, an NRF2 inhibitor, suppressed NRF2 activity in both cell lines (Figure 1A). These results highlight the difference in NRF2 signaling regulation between these two cell lines and suggest the usefulness of these two reporter platforms for identifying selective NRF2 regulators. 

**Figure 2:**
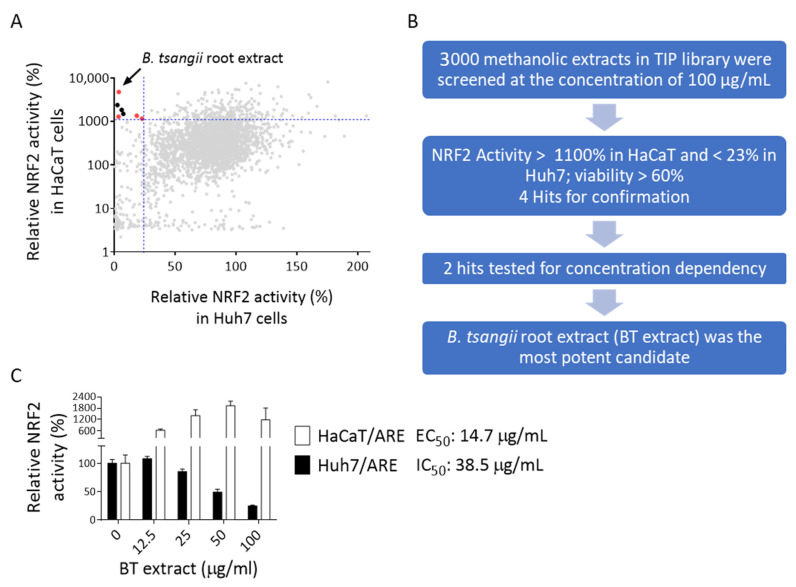
Identification of *Beilschmiedia tsangii* root extract as a selective NRF2 regulator. (**A**) The XY scatterplot shows the distribution of relative NRF2 activity in primary screening. Extracts that induced NRF2 activity to >1100% (horizontal dashed line, which was approximately equivalent to mean + 1SD of all tested extracts) in HaCaT/ARE cells and reduced NRF2 activity in Huh7/ARE cells to <23% (vertical dashed line, which was approximately equivalent to mean—2SD of all tested extracts) without severe cytotoxicity (>60% of DMSO control wells) were selected as hits of the primary screen (red dots). The screening results of *Beilschmiedia tsangii* root extract (BT extract) are indicated. (**B**) Screening flowchart for the identification of selective regulators of NRF2. (**C**) Huh7/ARE and HaCaT/ARE cells were treated with indicated concentrations of BT extracts for 24 h. NRF2 activity was calculated as described in Figure 1D.

The qualities of these two platforms were evaluated by signal-to-background ratio (S/B), signal-to-noise ratio (S/N), and Z’-factor. The S/N, S/B, and Z’-factor for Huh7/ARE platform were 7.8, 24.1, and 0.69, respectively, and those for HaCaT/ARE platform were 10.5, 77.7, and 0.64, respectively (Figure 1D and Table 1). These results suggest that the assay conditions of these platforms can be used for high-throughput screening (HTS). To identify selective NRF2 regulators, we performed HTS of the Taiwanese Indigenous Plant (TIP) Extract Library (3000 extracts) [20] using these two NRF2 reporter platforms. The screening was performed as described in Section 2. Extracts that could reduce NRF2 activity in Huh7 cells to 23% or below without severe cytotoxicity (> 60% viability compared to DMSO control wells) and increase NRF2 activity in HaCaT cell to 1100% or above were considered as hits in primary screening. A total of four extracts were selected for validation, and two extracts that passed validation were further tested in three concentrations (Figure 2A,B). *Beilschmiedia tsangii* Merr. (Lauraceae) root extract (BT extract) was the most potent candidate, with IC_50_ of 38.5 μg/mL in NRF2 inhibition in Huh7 cells and EC_50_ of 14.7 μg/mL in NRF2 activation in HaCaT cells (Figure 2C). 

### 3.2. Potential Active Ingredients in BT Extracts

**Figure 3:**
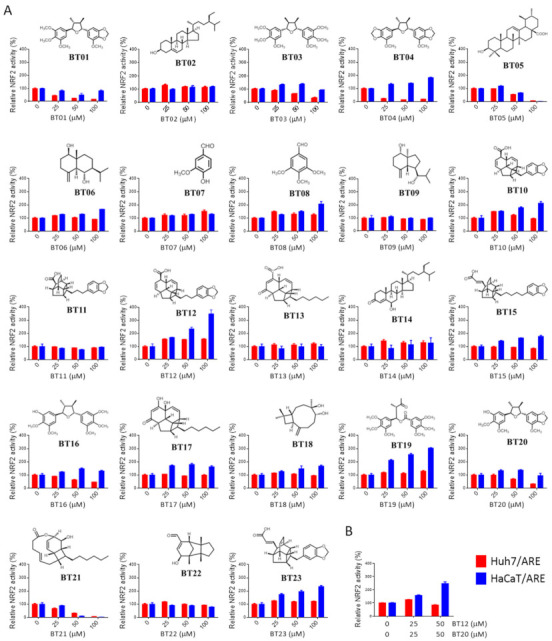
Potential active ingredients in BT extracts. (**A**) Concentration-response plots of compounds isolated from *B. tsangii*. The Huh7/ARE and HaCaT/ARE cells were treated with 23 compounds isolated from *B. tsangii* at 25, 50, and 100 μM for 24 h. The relative NRF2 activity was determined as described in Figure 1D. Data are presented as mean ± SD, *n* = 3. The compound names are listed in Supplementary Table S1. (**B**) Combinatory effects of BT12 and BT20 on NRF2 activity in Huh7 and HaCaT cells. The Huh7/ARE and HaCaT/ARE cells were treated with BT12 and BT20 at 25 and 50 μM for 24 h. Then, the relative NRF2 activity was determined.

The chemical constituents of the root of *B. tsangii* have been reported previously [24,25]. We evaluated the effects of 23 compounds (Supplementary Table S1), including endiandric acid analogs (BT10–13, 15, 17, 21, 23), tetrahydrofuran-type lignans (BT01, 03, 04, 16, and 20), benzenoids (BT07, 08, and 19), sesquiterpenes (BT06, 09, 18, and 22), triterpene (BT05), and steroids (BT02 and 14), on NRF2 activity in both Huh7/ARE and HaCaT/ARE cells. The results showed that BT04 is the only compound that can suppress NRF2 activity in Huh7 cells while enhancing it in HaCaT cells (Figure 3A). However, the NRF2 activity induction efficacy (182% at 100 μM) of BT04 was not comparable to that of BT extract (1179% at 100 μg/mL). Interestingly, we found that these compounds can be grouped into different types according to their effects on NRF2 activity in these two cell lines. Firstly, all lignans (BT01, 03, 04, 16, and 20) displayed Huh7-cell-specific NRF2 inhibitory effects to different extents. Secondly, BT19 and some endiandric acid analogs (BT10, 12, and 23) caused notable enhancement of NRF2 activity (> 200% at 100 μM) specifically in HaCaT cells. Lastly, BT05 and BT21 inhibited NRF2 activity in both cell lines. We next tested whether the combination of different types of compounds could recapitulate the dual-selective effect of BT extract on these two cell lines. Due to the availability of these 23 compounds, we only evaluated the combinatory effect of BT12, a HaCaT-specific NRF2 enhancer, and BT20, a Huh7-specific NRF2 inhibitor. The result clearly demonstrated that the combination treatment of BT12 and BT20 displayed a dual-selective NRF2 regulatory activity in HaCaT and Huh7 cells (Figure 3B). These findings strongly suggest that the dual selectivity of BT extract on NRF2 signaling in normal and cancer cells is most likely contributed by multiple components/mechanisms.

### 3.3. Effects of BT Extract on NRF2 Signaling in Huh7 and HaCaT Cells

**Figure 4:**
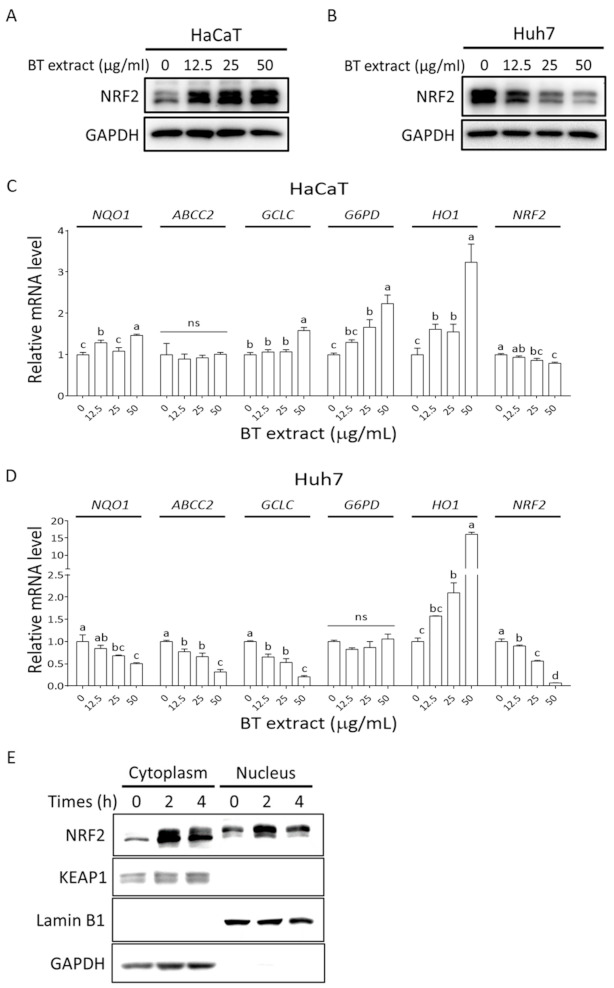
The effect of BT extract on NRF2 signaling in Huh7 and HaCaT cells. (**A**,**B**) HaCaT and Huh7 cells were treated with indicated concentrations of BT extracts for 8 h. NRF2 protein level was measured by immunoblot. (**C**,**D**) The HaCaT and Huh7 cells were treated with indicated concentrations of BT extracts for 18 h. Total RNA was isolated and submitted to reverse transcription and QPCR for the detection of NRF2 and expression levels of its target genes. Statistical analysis was performed using ANOVA, and different superscript letters indicate statistically significant differences (*p* < 0.05); ns, nonsignificant. (**E**) HaCaT cells were exposed to 50 μg/mL BT extract for 2 and 4 h. The levels of NRF2 and KEAP1 in cytoplasm and nuclei were detected by immunoblot. Lamin B1 and GAPDH served as nuclear and cytoplasmic markers, respectively.

Next, we evaluated the effect of BT extract on NRF2 signaling. BT extract increased NRF2 protein level in a concentration-dependent manner in HaCaT cells, while reducing it in Huh7 cells (Figure 4A,B). Moreover, a significant and concentration-dependent reduction in NRF2 mRNA was observed in BT-extract-treated Huh7 cells. Interestingly, BT extract also caused a slight yet significant decrease in NRF2 mRNA level in HaCaT cells (Figure 4C,D). These results indicate that BT extract could suppress NRF2 expression at the transcription level via a mechanism that is more robust in Huh7 cells than in HaCaT cells. In addition, these findings also suggest that BT extract increased NRF2 protein expression post-transcriptionally in HaCaT cells. To attain a deeper understanding of the effects of BT extract on NRF2 activation in HaCaT cells, we isolated cytoplasmic and nuclear proteins and analyzed the expression levels of NRF2 and KEAP1 in both fractions. The results showed that treatment with BT extract significantly increased both cytoplasmic and nuclear NRF2 protein expression in HaCaT cells within 2 h and still maintained a higher level of expression after 4 h, while neither the protein level nor the location of KEAP1 was affected by BT extract treatment. NRF2 showed faster and slower mobility bands in immunoblotting. Interestingly, while the slower mobility band appeared mainly in the nuclear fraction in the DMSO control group, its expression increased substantially in both cytoplasmic and nuclear fractions upon BT extract treatment (Figure 4E). These results suggest that BT extract could induce NRF2 protein accumulation and alterations in its posttranslational modification, which in turn promote the nuclear translocation of NRF2. Next, the mRNA expression levels of NRF2 target genes, namely NQO1, ABCC2, GCLC, G6PD, and HO1, were determined by QPCR. BT extract induced significant and concentration-dependent induction of G6PD and HO1 expression in HaCaT cells. NQO1 and GCLC were only slightly induced by BT extract, and ABCC2 did not seem to be affected by BT extract in HaCaT cells (Figure 4C). On the other hand, BT extract caused a significant concentration-dependent reduction in NQO1, ABCC2, and GCLC expression in Huh7 cells (Figure 4D). No notable change in G6PD expression in Huh7 cells was observed upon BT extract treatment. Surprisingly, a significant induction of HO1 expression was found in BT-extract-treated Huh7 cells (Figure 4D). To obtain further insight into this phenomenon, Huh7/ARE cells bearing shSCR or shNRF2 were treated with BT extract and used for HO1 expression measurement. We found that BT extract dramatically induced HO1 expression in NRF2-depleted Huh7 cells (Figure S4). This finding suggests that BT extract could suppress NRF2 signaling in Huh7 cells, while activating it in HaCaT cells, and that BT-extract-mediated HO1 induction in Huh7 cells mostly relies on NRF2-independent mechanism(s).

### 3.4. BT Extract Sensitizes Huh7 Cell to Sorafenib Treatment

**Figure 5:**
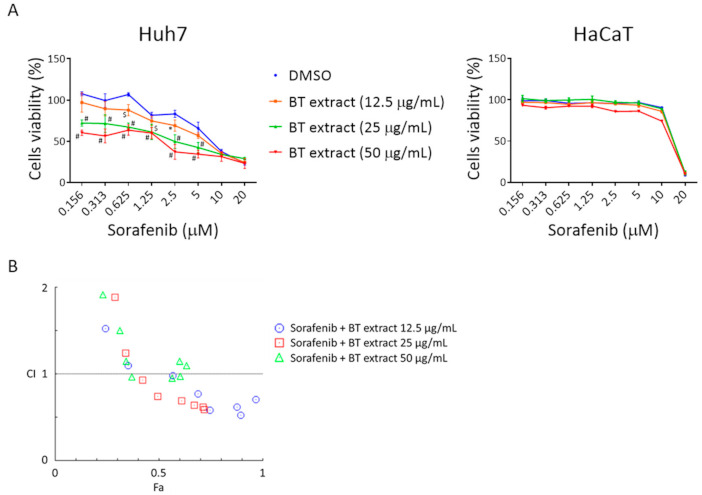
BT extract selectively potentiates sorafenib-mediated cytotoxicity in Huh7 cells. (**A**) Huh7 and HaCaT cells were exposed to indicated concentrations of sorafenib in the absence or presence of indicated concentrations of BT extracts for 72 h. The alamarBlue reagent was used to determine cell viability. DMSO solvent control was used as a standard for 100% activity. Data are presented as mean ± SD from three independent experiments. Statistical analysis was performed with results from Huh7 set using two-way ANOVA followed by Dunnett’s multiple comparisons test: * *p* < 0.05; ^$^ *p* < 0.001; ^#^ *p* < 0.0001 vs. DMSO group. (**B**) Combination index (CI) for sorafenib and BT extract in Huh7 cells was calculated using CompuSyn software.

Inhibition of NRF2 signaling has been reported to make cancer cells vulnerable to anticancer drugs [7,12]. Thus, we tested whether BT extract could sensitize Huh7 cells to sorafenib. Combination of BT extract and sorafenib induced more substantial cytotoxicity effects in Huh7 cells than those of sorafenib alone (Figure 5A). We further evaluated the effects of treatment with sorafenib and BT extract by determining the combination index (CI) values using the Chou–Talalay method [26]. CI < 1 indicates synergistic effect, while CI = 1 and CI > 1 indicate additive and antagonistic effects, respectively. A slight to moderate synergistic effect was observed in some combinations of sorafenib with 12.5 or 25 μg/mL BT extracts (Figure 5B and Table 2). On the other hand, we hardly detected any change in sorafenib-induced cytotoxicity in HaCaT cells with or without the presence of BT extract (Figure 5). These findings suggest that BT extract selectively sensitized Huh7 cells to sorafenib. 

### 3.5. BT Extract Reduces Sorafenib-Mediated Expression of Hyperkeratotic Factors in HaCaT Cells

**Figure 6:**
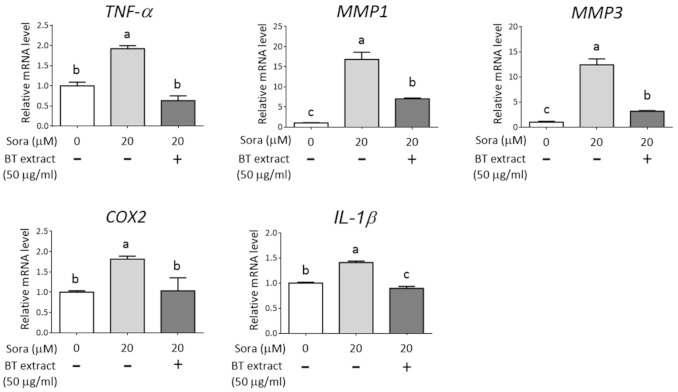
BT extract reduces the expression of hyperkeratotic factors caused by sorafenib in HaCaT cells. HaCaT cells were pretreated with or without BT extract (50 μg/mL) for 8 h and then exposed to indicated concentrations of Sora for 18 h. Total RNA was submitted to reverse transcription and real-time PCR for the detection of *TNF*-α, *COX2*, *IL-1β*, *MMP1*, and *MMP3* genes. For normalization, *GAPDH* was analyzed as an internal control. Data are expressed as relative mRNA level to nontreated group and presented as mean ± SD from three independent experiments. Statistical analysis was performed using ANOVA, and different superscript letters indicate statistically significant differences (*p* < 0.05).

Hand–foot skin reaction (HFSR) is one of the most common adverse effects of sorafenib. However, the mechanism of the cutaneous toxicity remains largely unknown, and optimal treatment or prevention has yet to be developed [17,27]. A main feature of HFSR is hyperkeratosis, which is also frequently observed in patients who suffer from adverse effects induced by tyrosine kinase inhibitors. Upregulation of TNF-α has been detected in such skin lesions [28]. Genetic polymorphism and serum level of TNF-α have been shown to be associated with sorafenib-induced HFSR [29]. TNF-α can induce inflammation and participate in the impairment of skin barrier function, which thus increases the sensitivity of skin to environmental insults [28,30]. Abnormal expression of matrix metalloproteinases such as MMP1 and MMP3 is known to affect the homeostasis of keratinocytes [31,32,33]. In addition, MMP1 and MMP3 can be induced by TNF-α and sorafenib treatment, respectively [34,35]. Moreover, these genes have been shown to be downregulated by NRF2 signaling [36,37,38]. Here, we found that sorafenib can induce mRNA expression of *TNF-α*, *COX2*, *IL-1β*, *MMP1*, and *MMP3* in HaCaT cells. More importantly, BT extract significantly reversed sorafenib-mediated induction of these genes (Figure 6). This finding suggests a potential beneficial effect of BT extract on sorafenib-induced skin toxicity.

## 4. Discussion

NRF2, a pleiotropic transcription factor, not only plays a crucial role in cancer prevention but also promotes each hallmark of cancer through direct or indirect manners [39]. With its dual roles in normal cells and cancer cells, modulation of NRF2 activity should be performed cautiously. Ideally, a selective NRF2 inhibitor would suppress NRF2 signaling only in cancer cells but not in normal cells; thus, it could enhance the therapeutic effects of anticancer drugs without worsening side effects. By contrast, a selective NRF2 activator would only activate NRF2 signaling in normal cells; therefore, it could ameliorate the adverse effects of anticancer drugs without compromising therapeutic effects. Moreover, we wondered if an NRF2 regulator could possess dual selectivity in cancer cells and normal cells. Therefore, a combination of platforms to identify selective NRF2 regulators is required for the development of novel therapeutic modalities targeting NRF2 signaling. Here, we used a liver cancer cell line, in which NRF2 signaling is consistently activated, and a normal keratinocyte cell line as models, and we built stable screening platforms to monitor the effects of plant extracts on NRF2 activities in both cell lines. Interestingly, an extract of the root of *Beilschmiedia tsangii* (BT extract) was identified to display opposite selective effects, in which BT extract suppressed NRF2 signaling in an NRF2-hyperactivated liver cancer cell line and induced NRF2 signaling in a normal keratinocyte cell line. More importantly, we showed that BT extract caused cell-specific alterations in NRF2 mRNA and protein levels in Huh7 and HaCaT cells. The results of our study suggest that these platforms could represent a novel strategy to identify single/dual-selective NRF2 regulators. 

In general, in our platforms, tBHQ increases NRF2 activity to around 850–1100% in HaCaT cells and luteolin reduces NRF2 activity to around 7–21% in Huh7 cells when compared to their own DMSO controls (100%) (Figure 1D). The screening results showed that the average effects of these 3000 extracts were around 463% and 76% in HaCaT and Huh7 cells, respectively. These findings indicate that a majority of extracts in the TIP library exert mild NRF2-activating effects in HaCaT cells. Moreover, among 3000 extracts, there were 239 (~8%) extracts that showed activity greater than 1100% and could be considered as substantial NRF2 inducers in HaCaT cells. On the other hand, there were 126 (4.2%) extracts that reduced NRF2 activity to less than 23% of DMSO controls in Huh7 cells. However, only 4 out of 3000 extracts fit both criteria and were selected for further validation tests. Among them, only two extracts passed the tests and were considered as hits of this high-throughput screening. These results clearly show the scarceness of extracts with dual selectivity. It is also worth noting that 9 extracts among the 239 substantial NRF2 inducers in HaCaT cells could also enhance NRF2 activity to 150–200% in Huh7 cells. Although the chance of identifying cancer cell/normal cell nonselective NRF2 activators might be limited, our screening results showed that there are still plant extracts that could further induce NRF2 activity even in NRF2-hyperactivated cancer cells. On the other hand, 80 extracts out of the 126 NRF2 suppressors in Huh7 cells could also reduce NRF2 activity to less than 23% in HaCaT cells. These results suggest that using NRF2-hyperactivated cancer cells as a platform to screen NRF2 inhibitors could possibly generate a majority of hits that also suppress NRF2 activity in normal cells. Accordingly, these results further demonstrate the advantage of utilizing NRF2-hyperactivated cancer cells and normal cells simultaneously as selective NRF2 regulator screening platforms. 

As aforementioned, NRF2 has been considered as a therapeutic target for cancer treatment. Many synthetic and natural compound NRF2 inhibitors have been discovered and evaluated in vitro and in vivo [11,12]. However, some NRF2 inhibitors, such as luteolin, apigenin, and 4-methoxychalcone, display mixed effects on NRF2 signaling. Depending on treatment concentration or cell type, these compounds could either activate or inhibit NRF2 signaling [11]. One possible explanation for the divergent effects of NRF2 inhibitors is that aberrant NRF2 activation can be caused by multiple mechanisms, including genetic mutations, epigenetic changes, transcriptional alterations, metabolic changes, and altered interaction with regulators [8,12]. Moreover, NRF2 activation may be beneficial not only in reducing adverse effects but also in enhancing cancer immunotherapy via inhibiting the immunosuppressive and tumor-promoting functions of myeloid-derived suppressor cells (MDSCs) [40,41]. Thus, NRF2 activators could be considered as an immunostimulatory therapy for cancer. One argument is that the effect of NRF2 activators on cancer cells with persistent NRF2 activation is negligible because NRF2 signaling is already strongly activated in these cancer cells [8,41]. Our results for tBHQ treatment also support this notion (Figure 1A). However, one should also notice that several extracts could still increase NRF2 activity in Huh7 cells to more than 150% (Figure 2A). To take all these observations into consideration, and also from a precision medicine point of view, a panel of different cancer cell platforms and platform pairs with different noncancer cells are required to profile the cell-context-dependent effects of each NRF2 regulator. Therefore, identifying or building up the most representative cell line or cell line panel for evaluating the effects of NRF2 regulators in different cell contexts would be an interesting and important task to be completed in the future.

In the screening of pure compounds isolated from the root of *B. tsangii*, we found that only one compound (BT04) displayed dual selectivity (Figure 3). However, the effect of BT04 on HaCaT cells was not comparable to that of BT extract. The simplest explanation is that the true major active component was not in the panel of compounds that we tested. These findings also imply a scarcity of compounds with dual selectivity. Nevertheless, we observed that two series of compounds showed opposite selectivity on NRF2 signaling: all lignans (BT01, 03, 04, 16, and 20) displayed Huh7-cell-specific NRF2 inhibitory effects, while three endiandric acid analogs (BT10, 12, and 23) caused HaCaT-cell-specific enhancement of NRF2 activity. These findings strongly suggest that the dual selectivity of BT extract on NRF2 signaling in HaCaT cells and Huh7 cells is most likely contributed by multiple components and different mechanisms. Based on this observation, we infer that a combination of normal-cell-specific NRF2 activators and cancer-cell-specific NRF2 inhibitors could represent a novel strategy to achieve selective regulation of NRF2 signaling for better cancer treatment. Nevertheless, it is also interesting to test whether the combination of two functional moieties, one each from selective NRF2 inhibitor and activator, would still possess dual selectivity on NRF2 signaling. Incidentally, some of the 23 compounds used in this study had been evaluated for their iNOS-inhibitory activities [24,25]. However, most of them displayed IC_50_ values of around 50 μM and did not correlate with NRF2 regulatory activities. 

As mentioned above, our inference is that multiple compounds contribute to the dual selectivity of BT extract. However, in fact, this is the fundamental of traditional herbal medicine: more than one component contributes to the overall therapeutic effect, and some components may not have therapeutic activities but are required for the combinatory effects [42,43]. To possess dual-selective NRF2 regulation function in cells with different regulatory contexts of the NRF2 pathway, the candidate must have the ability to modulate at least two distinct mechanisms. Thus, the conventional “one drug, one target, one mechanism” concept would not fit our screening described in this study. Moreover, chronic diseases such as cancer tend to stem from multiple molecular abnormalities [44]. Thus, a “multiple drugs, multiple targets” approach would be a more effective therapeutic strategy [45], which is the foundation of our screening principle. Furthermore, our results also highlight the advantage of utilizing plant extracts or partially purified fractions for the discovery of selective NRF2 regulators. Finally, we are still working on the expansion of the Taiwanese Indigenous Plant Extract Library. We believe that this library, which has high chemical diversity, is a useful resource for new drug development.

## Figures and Tables

**Table 1:** Statistical parameters of screening platform.

Parameters	Values in HaCaT/ARE	Values in Huh7/ARE
S/B ^a^	10.5	7.8
S/N ^a^	77.7	24.1
Z’-factor ^a^	0.64	0.69

^a^ Parameters were calculated as follows: S/B = mean of the maximum signal/mean of the minimum signal; S/N = |mean of signal − mean of background|/SD (standard deviation) of background; Z’ = 1 − (3SD of positive control + 3SD of negative control)/|mean of positive control − mean of negative control|. DMSO was considered as background, while luteolin- and tBHQ-treated wells were defined as signal for S/N calculation. Luteolin- and tBHQ-treated wells were defined as positive controls and DMSO was defined as negative control for calculating Z’-factor.

**Table 2:** The combination index (CI) of sorafenib and BT extract treatment on Huh7 cells.

Sorafenib (μM)	BT Extract (μg/mL)		
	12.5	25	50
20.000	1.522	1.885	1.917
10.000	1.095	1.242	1.507
5.000	0.985	0.930	1.150
2.500	0.773	0.740	0.964
1.250	0.587	0.689	1.150
0.625	0.618	0.639	1.100
0.313	0.524	0.617	0.955
0.156	0.708	0.588	0.977

## Data Availability

Not applicable.

## Supplementary Materials

The following are available online at https://www.mdpi.com/article/10.3390/antiox10040544/s1, Figure S1: Basal NRF2 expression level and activity in different cell lines, Figure S2: Construction of lentiviral-based NRF2 luciferase reporter plasmid, Figure S3: Validation of shRNA-mediated NRF2 knockdown, Figure S4: BT-extract-mediated HO1 induction was NRF2-independent, Figures S5–7: Raw images of all Western blot results, Table S1: Compounds used in this study.

## References

[1] Jemal A., Bray F., Center M.M., Ferlay J., Ward E., Forman D. (2011). Global cancer statistics. CA Cancer J. Clin..

[2] Zhai B., Sun X.Y. (2013). Mechanisms of resistance to sorafenib and the corresponding strategies in hepatocellular carcinoma. World J. Hepatol..

[3] Jiang T., Harder B., Rojo de la Vega M., Wong P.K., Chapman E., Zhang D.D. (2015). p62 links autophagy and Nrf2 signaling. Free Radic. Biol. Med..

[4] Ruiz S., Pergola P.E., Zager R.A., Vaziri N.D. (2013). Targeting the transcription factor Nrf2 to ameliorate oxidative stress and inflammation in chronic kidney disease. Kidney Int..

[5] Kim H.J., Vaziri N.D. (2010). Contribution of impaired Nrf2-Keap1 pathway to oxidative stress and inflammation in chronic renal failure. Am. J. Physiol. Ren. Physiol..

[6] Hayes J.D., McMahon M., Chowdhry S., Dinkova-Kostova A.T. (2010). Cancer chemoprevention mechanisms mediated through the Keap1-Nrf2 pathway. Antioxid. Redox Signal..

[7] Menegon S., Columbano A., Giordano S. (2016). The Dual Roles of NRF2 in Cancer. Trends Mol. Med..

[8] Kitamura H., Motohashi H. (2018). NRF2 addiction in cancer cells. Cancer Sci..

[9] Choi B.-h., Kwak M.-K. (2016). Shadows of NRF2 in cancer: Resistance to chemotherapy. Curr. Opin. Toxicol..

[10] Wu S., Lu H., Bai Y. (2019). Nrf2 in cancers: A double-edged sword. Cancer Med..

[11] Zhu J., Wang H., Chen F., Fu J., Xu Y., Hou Y., Kou H.H., Zhai C., Nelson M.B., Zhang Q. (2016). An overview of chemical inhibitors of the Nrf2-ARE signaling pathway and their potential applications in cancer therapy. Free Radic. Biol. Med..

[12] Panieri E., Saso L. (2019). Potential Applications of NRF2 Inhibitors in Cancer Therapy. Oxidative Med. Cell. Longev..

[13] Rojo de la Vega M., Dodson M., Chapman E., Zhang D.D. (2016). NRF2-targeted therapeutics: New targets and modes of NRF2 regulation. Curr. Opin. Toxicol..

[14] Singh A., Venkannagari S., Oh K.H., Zhang Y.Q., Rohde J.M., Liu L., Nimmagadda S., Sudini K., Brimacombe K.R., Gajghate S. (2016). Small Molecule Inhibitor of NRF2 Selectively Intervenes Therapeutic Resistance in KEAP1-Deficient NSCLC Tumors. ACS Chem. Biol..

[15] Manohar S., Leung N. (2018). Cisplatin nephrotoxicity: A review of the literature. J. Nephrol..

[16] Songbo M., Lang H., Xinyong C., Bin X., Ping Z., Liang S. (2019). Oxidative stress injury in doxorubicin-induced cardiotoxicity. Toxicol. Lett..

[17] Ai L., Xu Z., Yang B., He Q., Luo P. (2019). Sorafenib-associated hand-foot skin reaction: Practical advice on diagnosis, mechanism, prevention, and management. Expert Rev. Clin. Pharm..

[18] Negrette-Guzman M. (2019). Combinations of the antioxidants sulforaphane or curcumin and the conventional antineoplastics cisplatin or doxorubicin as prospects for anticancer chemotherapy. Eur. J. Pharm..

[19] Zhang D.D., Chapman E. (2020). The role of natural products in revealing NRF2 function. Nat. Prod. Rep..

[20] Yen C.H., Chang H.S., Yang T.H., Wang S.F., Wu H.C., Chen Y.C., Lin K.J., Wang S. (2018). High-Content Screening of a Taiwanese Indigenous Plant Extract Library Identifies Syzygium simile leaf Extract as an Inhibitor of Fatty Acid Uptake. Int. J. Mol. Sci..

[21] Li C.H., Yen C.H., Chen Y.F., Lee K.J., Fang C.C., Zhang X., Lai C.C., Huang S.F., Lin H.K., Arthur Chen Y.M. (2017). Characterization of the GNMT-HectH9-PREX2 tripartite relationship in the pathogenesis of hepatocellular carcinoma. Int. J. Cancer.

[22] Wu H.C., Cheng M.J., Yen C.H., Chen Y.A., Chen Y.S., Chen I.S., Chang H.S. (2020). Chemical Constituents with GNMT-Promoter-Enhancing and NRF2-Reduction Activities from Taiwan Agarwood Excoecaria formosana. Molecules.

[23] Zhang J.H., Chung T.D., Oldenburg K.R. (1999). A Simple Statistical Parameter for Use in Evaluation and Validation of High Throughput Screening Assays. J. Biomol. Screen..

[24] Huang Y.T., Chang H.S., Wang G.J., Cheng M.J., Chen C.H., Yang Y.J., Chen I.S. (2011). Anti-inflammatory endiandric acid analogues from the roots of Beilschmiedia tsangii. J. Nat. Prod..

[25] Huang Y.T., Chang H.S., Wang G.J., Lin C.H., Chen I.S. (2012). Secondary metabolites from the roots of Beilschmiedia tsangii and their anti-inflammatory activities. Int. J. Mol. Sci..

[26] Chou T.C. (2006). Theoretical basis, experimental design, and computerized simulation of synergism and antagonism in drug combination studies. Pharm. Rev..

[27] Vastarella M., Fabbrocini G., Sibaud V. (2020). Hyperkeratotic Skin Adverse Events Induced by Anticancer Treatments: A Comprehensive Review. Drug Saf..

[28] Pastore S., Lulli D., Girolomoni G. (2014). Epidermal growth factor receptor signalling in keratinocyte biology: Implications for skin toxicity of tyrosine kinase inhibitors. Arch. Toxicol..

[29] Lee J.H., Chung Y.H., Kim J.A., Shim J.H., Lee D., Lee H.C., Shin E.S., Yoon J.H., Kim B.I., Bae S.H. (2013). Genetic predisposition of hand-foot skin reaction after sorafenib therapy in patients with hepatocellular carcinoma. Cancer.

[30] Arrieta O., Carmona A., de Jesus Vega M.T., Lopez-Mejia M., Cardona A.F. (2016). Skin communicates what we deeply feel: Antibiotic prophylactic treatment to reduce epidermal growth factor receptor inhibitors induced rash in lung cancer (the Pan Canadian rash trial). Ann. Transl. Med..

[31] D’Armiento J., DiColandrea T., Dalal S.S., Okada Y., Huang M.T., Conney A.H., Chada K. (1995). Collagenase expression in transgenic mouse skin causes hyperkeratosis and acanthosis and increases susceptibility to tumorigenesis. Mol. Cell. Biol..

[32] Sevilla L.M., Latorre V., Sanchis A., Perez P. (2013). Epidermal inactivation of the glucocorticoid receptor triggers skin barrier defects and cutaneous inflammation. J. Investig. Derm..

[33] Mazzarella N., Femiano F., Gombos F., De Rosa A., Giuliano M. (2006). Matrix metalloproteinase gene expression in oral lichen planus: Erosive vs. reticular forms. J. Eur. Acad. Derm. Venereol..

[34] Yeo H., Lee J.Y., Kim J., Ahn S.S., Jeong J.Y., Choi J.H., Lee Y.H., Shin S.Y. (2020). Transcription factor EGR-1 transactivates the MMP1 gene promoter in response to TNFalpha in HaCaT keratinocytes. BMB Rep..

[35] Luo P., Yan H., Chen X., Zhang Y., Zhao Z., Cao J., Zhu Y., Du J., Xu Z., Zhang X. (2020). s-HBEGF/SIRT1 circuit-dictated crosstalk between vascular endothelial cells and keratinocytes mediates sorafenib-induced hand-foot skin reaction that can be reversed by nicotinamide. Cell Res..

[36] Yang D., Tan X., Lv Z., Liu B., Baiyun R., Lu J., Zhang Z. (2016). Regulation of Sirt1/Nrf2/TNF-alpha signaling pathway by luteolin is critical to attenuate acute mercuric chloride exposure induced hepatotoxicity. Sci. Rep..

[37] Zhao P., Alam M.B., Lee S.H. (2018). Protection of UVB-Induced Photoaging by Fuzhuan-Brick Tea Aqueous Extract via MAPKs/Nrf2-Mediated Down-Regulation of MMP-1. Nutrients.

[38] Fang W., Zhou X., Wang J., Xu L., Zhou L., Yu W., Tao Y., Zhu J., Hu B., Liang C. (2018). Wogonin mitigates intervertebral disc degeneration through the Nrf2/ARE and MAPK signaling pathways. Int. Immunopharmacol..

[39] Rojo de la Vega M., Chapman E., Zhang D.D. (2018). NRF2 and the Hallmarks of Cancer. Cancer Cell.

[40] Hiramoto K., Satoh H., Suzuki T., Moriguchi T., Pi J., Shimosegawa T., Yamamoto M. (2014). Myeloid lineage-specific deletion of antioxidant system enhances tumor metastasis. Cancer Prev. Res..

[41] Yen C.H., Hsiao H.H. (2018). NRF2 Is One of the Players Involved in Bone Marrow Mediated Drug Resistance in Multiple Myeloma. Int. J. Mol. Sci..

[42] Thomford N.E., Senthebane D.A., Rowe A., Munro D., Seele P., Maroyi A., Dzobo K. (2018). Natural Products for Drug Discovery in the 21st Century: Innovations for Novel Drug Discovery. Int. J. Mol. Sci..

[43] Wu C., Lee S.L., Taylor C., Li J., Chan Y.M., Agarwal R., Temple R., Throckmorton D., Tyner K. (2020). Scientific and Regulatory Approach to Botanical Drug Development: A U.S. FDA Perspective. J. Nat. Prod..

[44] Frantz S. (2005). Drug discovery: Playing dirty. Nature.

[45] Ahn K. (2017). The worldwide trend of using botanical drugs and strategies for developing global drugs. BMB Rep..

