# Supplementary material for: Identification of Beilschmiedia tsangii Root Extract as a Liver Cancer Cell–Normal Keratinocyte Dual-Selective NRF2 Regulator
Source: Antioxidants (Basel). 2021 Apr 1;10(4):544. doi: 10.3390/antiox10040544 (PMC8066689; doi:10.3390/antiox10040544)
Supplement: Supplementary file 1 [file antioxidants-10-00544-s001.pdf]

**Table S1. Compounds used in this study**	
**Code**	Name
BT01	*rel*-(7*R*,8*R*,7′*R*,8′*R*)-3′,4′-Methylenedioxy-3,4,5,5′-tetramethoxy-7,7′-epoxylignan
BT02	β-Sitosterol
BT03	*rel*-(7*S*,8*S*,7′*R*,8′*R*)-3,3′,4,4′,5,5′-Hexamethoxylignan
BT04	*rel*-(7*R*,8*R*,7′*R*,8′*R*)-3,4,3′,4′-Dimethylenedioxy-5,5′-dimethoxy-7,7′-epoxylignan
BT05	Ursolic acid
BT06	Eudesm-4(15)-ene-1β,6α-diol
BT07	Vanillin
BT08	3,4,5-Trimethoxybenzaldehyde
BT09	Octahydro-4-hydroxy-3α-methyl-7-methylene-α-(1-methylethyl)-1*H*-indene-1-methanol
BT10	Tsangibeilin B
BT11	Endiandric acid K
BT12	Tsangibeilin A
BT13	Tsangibeilin C
BT14	6β-Hydroxystigmast-4-en-3-one
BT15	Endiandric acid L
BT16	*rel*-(7*R*,8*R*,7′*R*,8′*R*)-3,3′,4,4′,5,5′-Hexamethoxylignan
BT17	Tsangibeilin D
BT18	(4*R*,5*R*)-4,5-Dihydroxycaryophyll-8(13)-ene
BT19	Tsangin C
BT20	Beilschminol A
BT21	Tricyclotsangibeilin
BT22	(+)-5-Hydroxybarbatenal
BT23	Endiandric acid M

Supplementary Figure S1

A

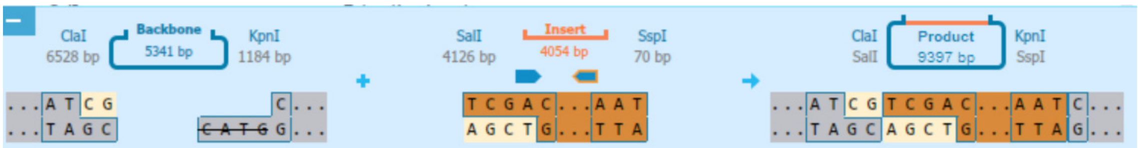

B

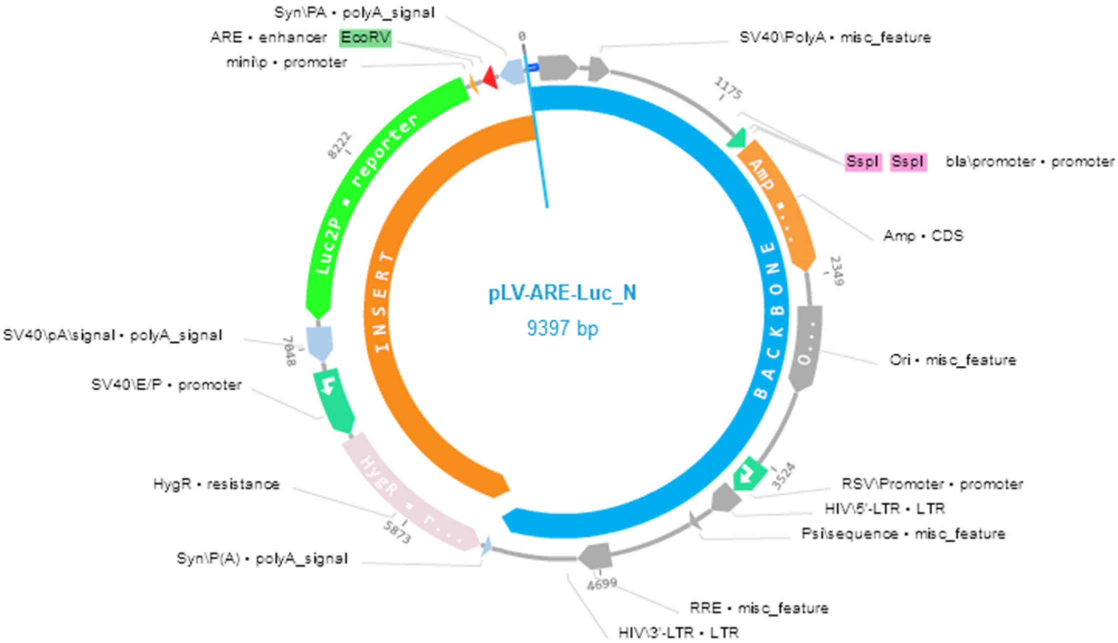

Supplementary Figure S1. Construction of lentiviral-based NRF2 luciferase reporter plasmid. (A) Concept of construction. A plasmid (clone # TRCN0000072249) purchased from RNAi Core was digested by *Cla*I and *Kpn*I, and the 5.3 Kb resultant fragment was used as backbone. The pGL4.37[luc2P/ARE/Hygro] plasmid (Promega) was digested by *Ssp*I and *Sal*I to generate the 4 Kb insert fragment that contains all required elements for determining NRF2 activity. Resultant lentiviral NRF2 reporter plasmid was generated by ligation of these two fragments. (B) The map of resultant plasmid, pLV-ARE-Luc_R. The orientation of insert was confirmed by restriction enzyme digestion with *Ssp*I and *Eco*RV.

Supplementary Figure S2

A

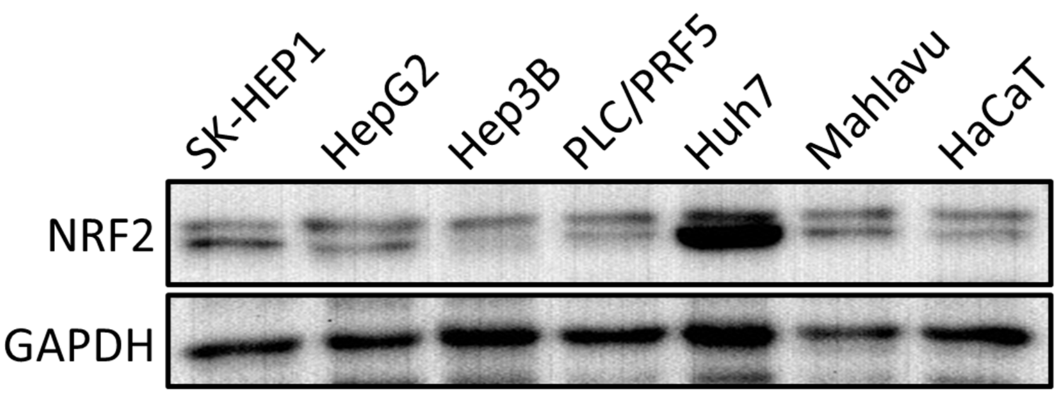

B

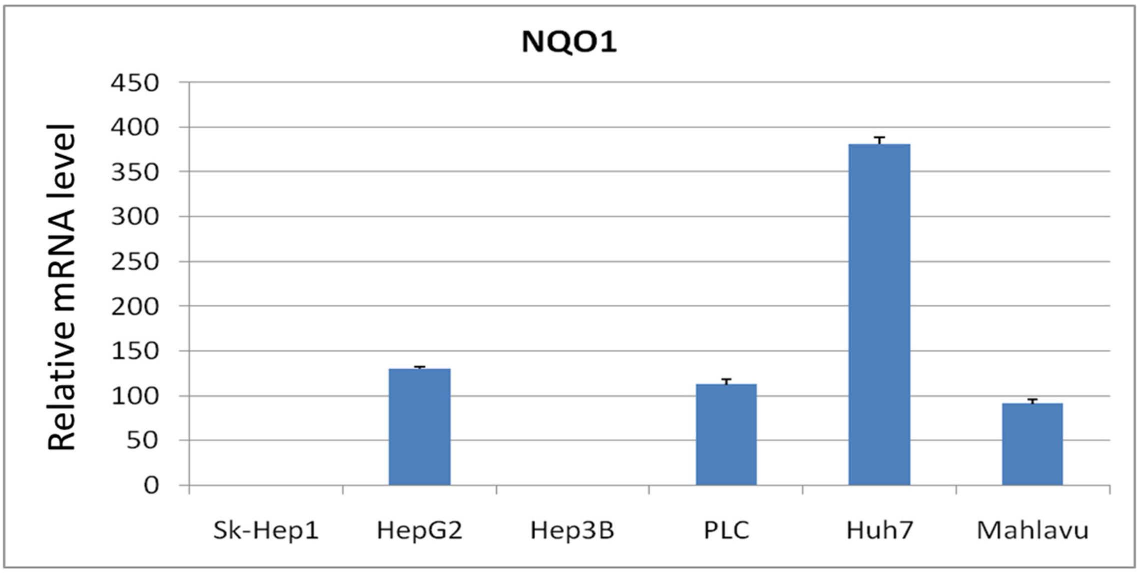

Supplementary Figure S2. Basal NRF2 expression level and activity in different cell lines. (A) The basal NRF2 protein expression levels were determined in 6 liver cancer cell lines, namely Sk-Hep1, HepG2, Hep3B, PLC/PRF/5, Huh7, and Mahlavu, and in 1 normal keratinocyte cell line, HaCaT. (B) The mRNA level of NRF2 target gene *NQO1* was determined by QPCR in 6 liver cancer cell lines.

Supplementary Figure S3

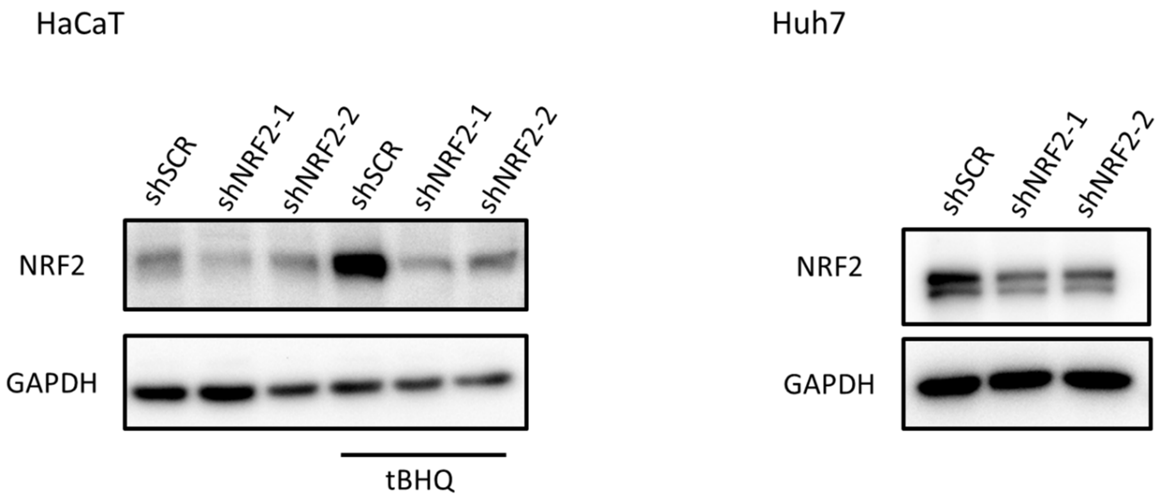

Supplementary Figure S3. Validation of shRNA-mediated NRF2 knockdown. The protein expression level of NRF2 was determined in HaCaT/ARE and Huh7/ARE stable reporter cells expressing two different NRF2 shRNAs (shNRF2-1 and shNRF2-2) or control scramble shRNA (shSCR). In addition, lysates from tBHQ (10 μM)-treated HaCaT/ARE stable reporter cells were analyzed as well.

Supplementary Figure S4.

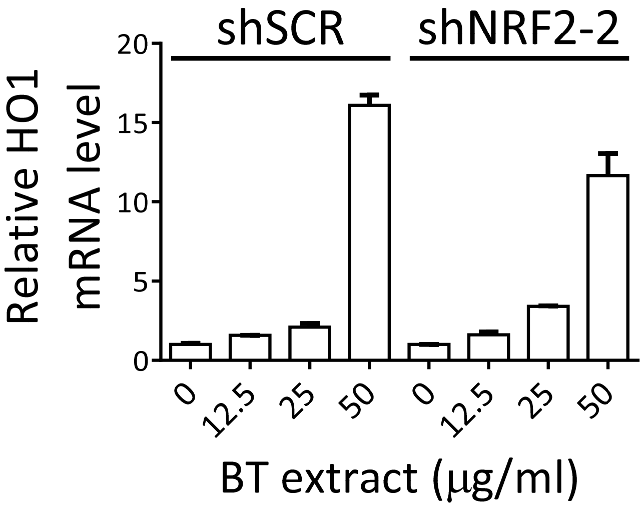

Supplementary Figure S4. BT-extract-mediated HO1 induction was NRF2-independent. The level of HO1 mRNA in Huh7/ARE cells expressing control scramble shRNA (shSCR) or NRF2 shRNA was determined by QPCR.

Supplementary Figure S5.

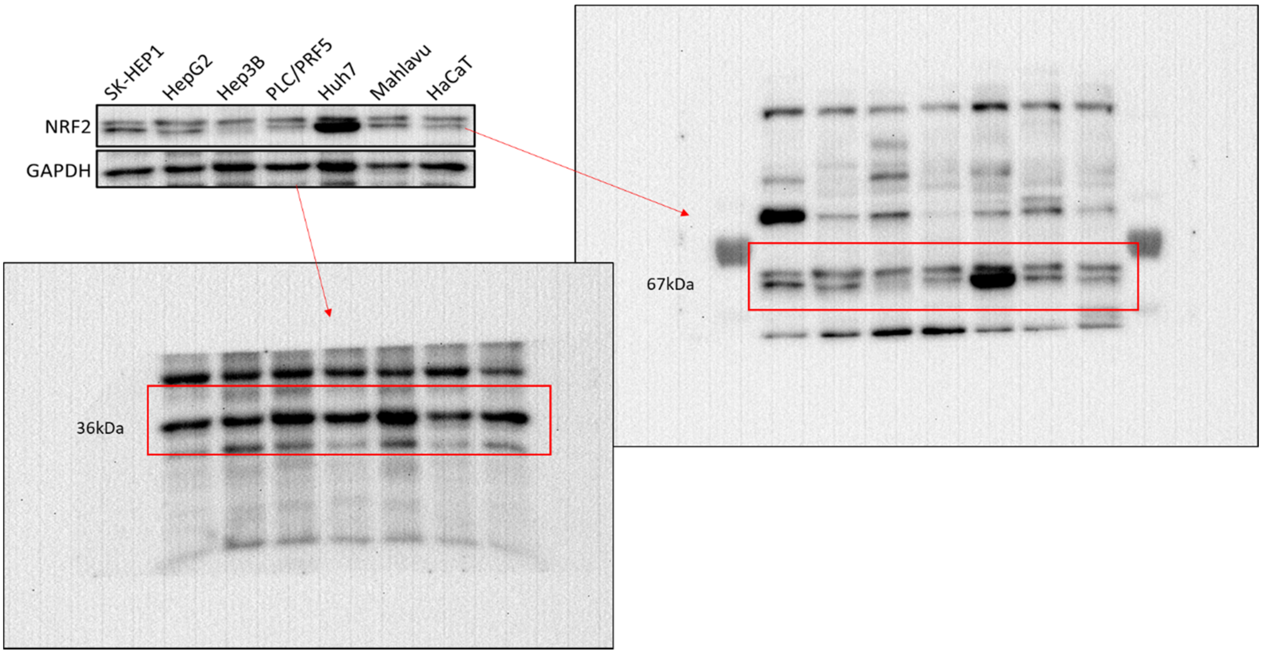

Supplementary Figure S5. Raw blot data related to Supplementary Figure S2. The NRF2 antibody used in this experiment was anti-NRF2 (sc-722, Santa Cruz Biotechnology, CA, USA).

Supplementary Figure S6.

A.

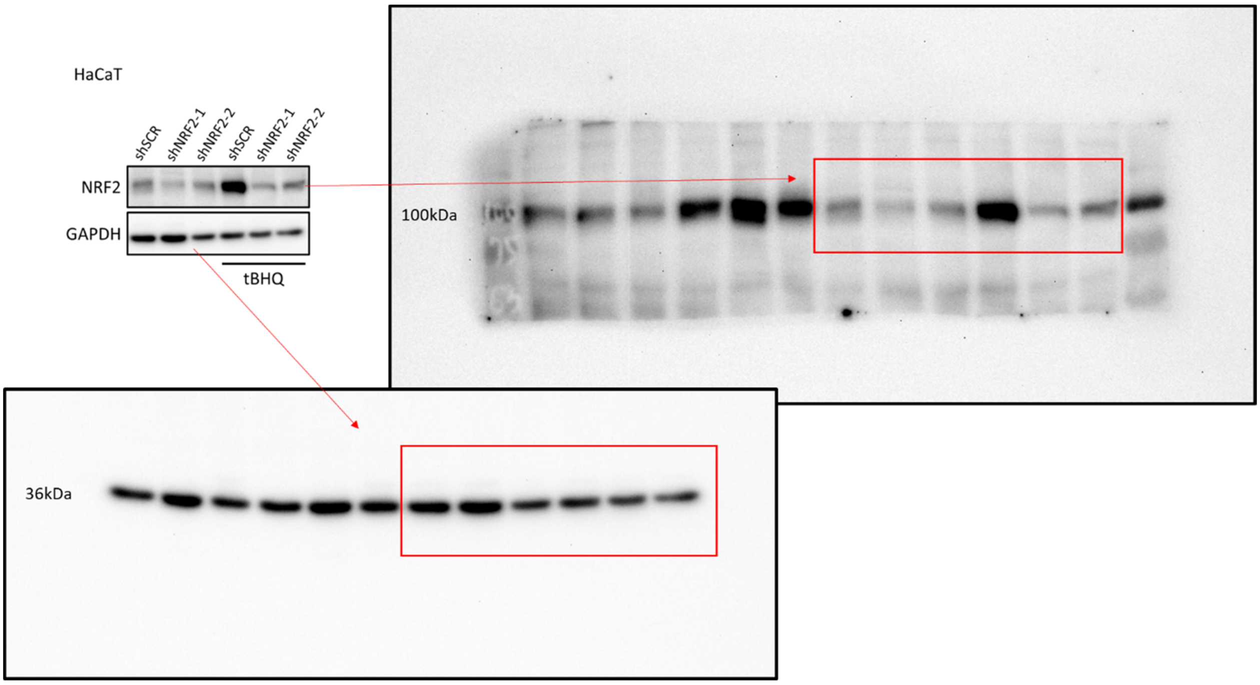

B.

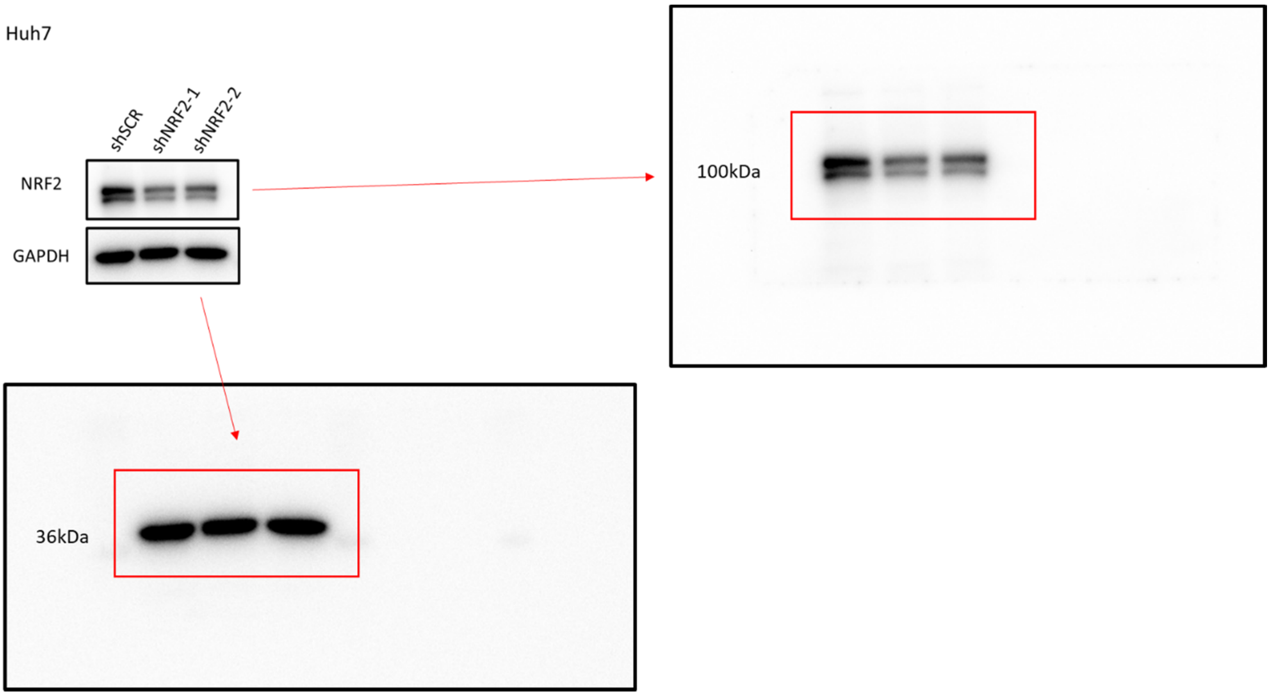

Supplementary Figure S6. Raw blot data related to Supplementary Figure S3.

Supplementary Figure S7.

A.

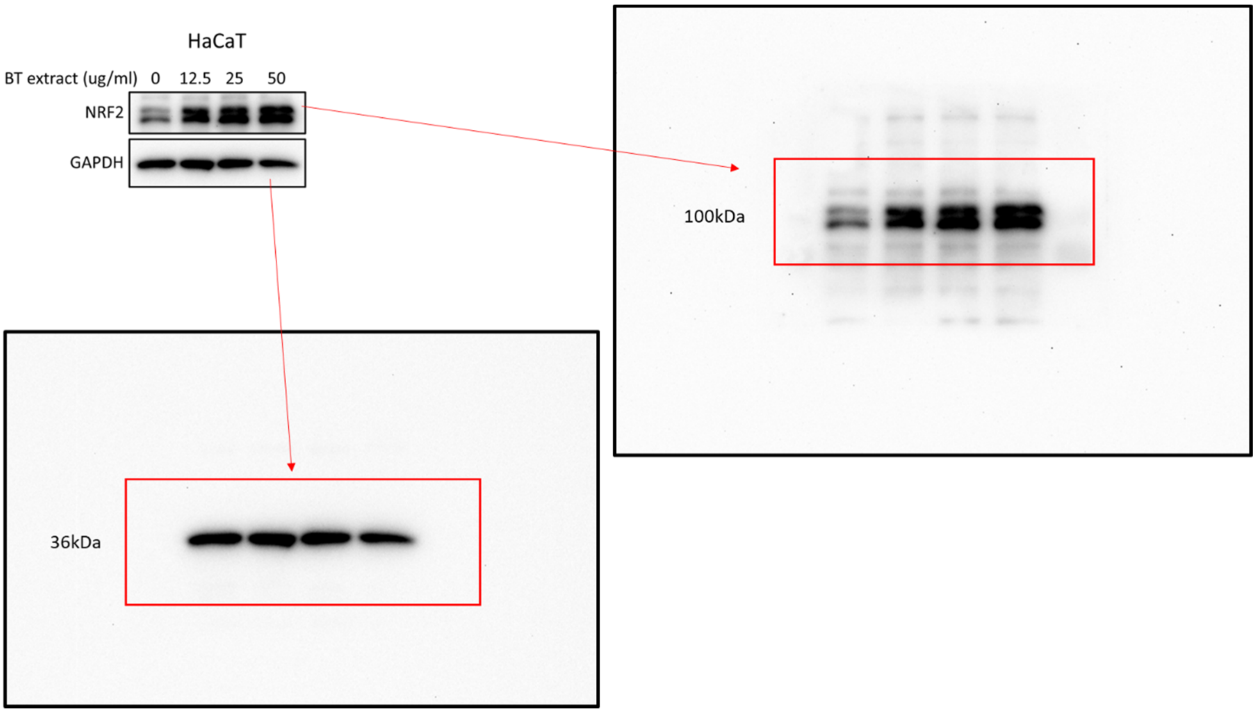

B.

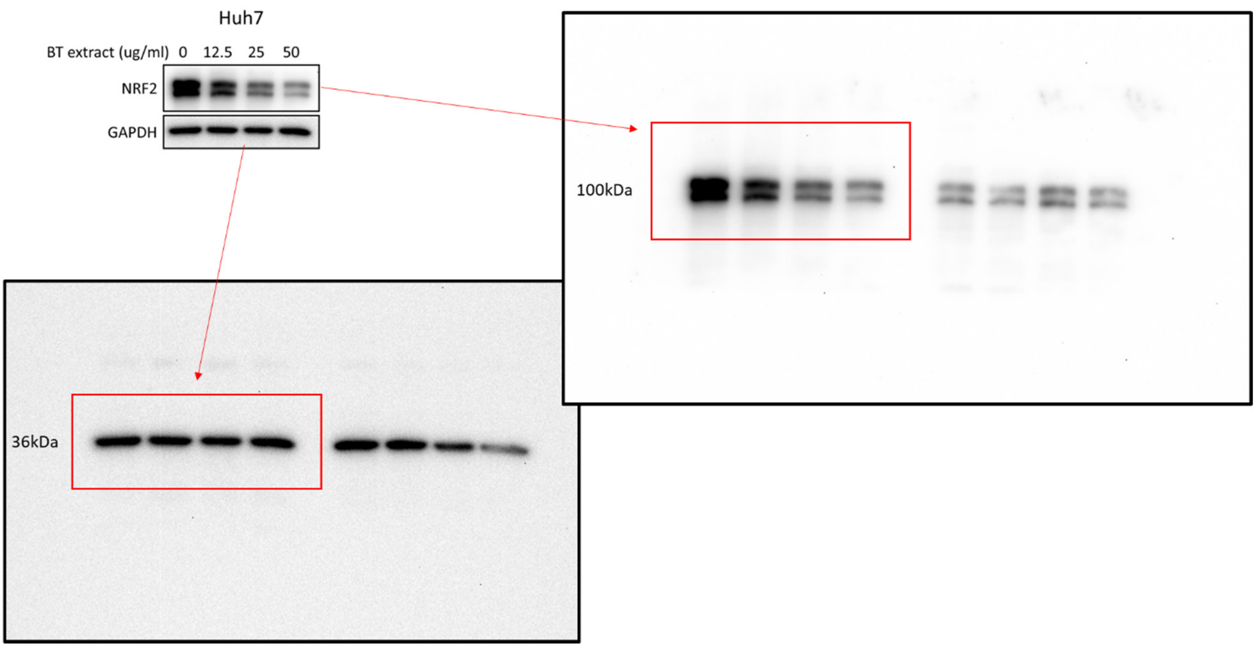

C.

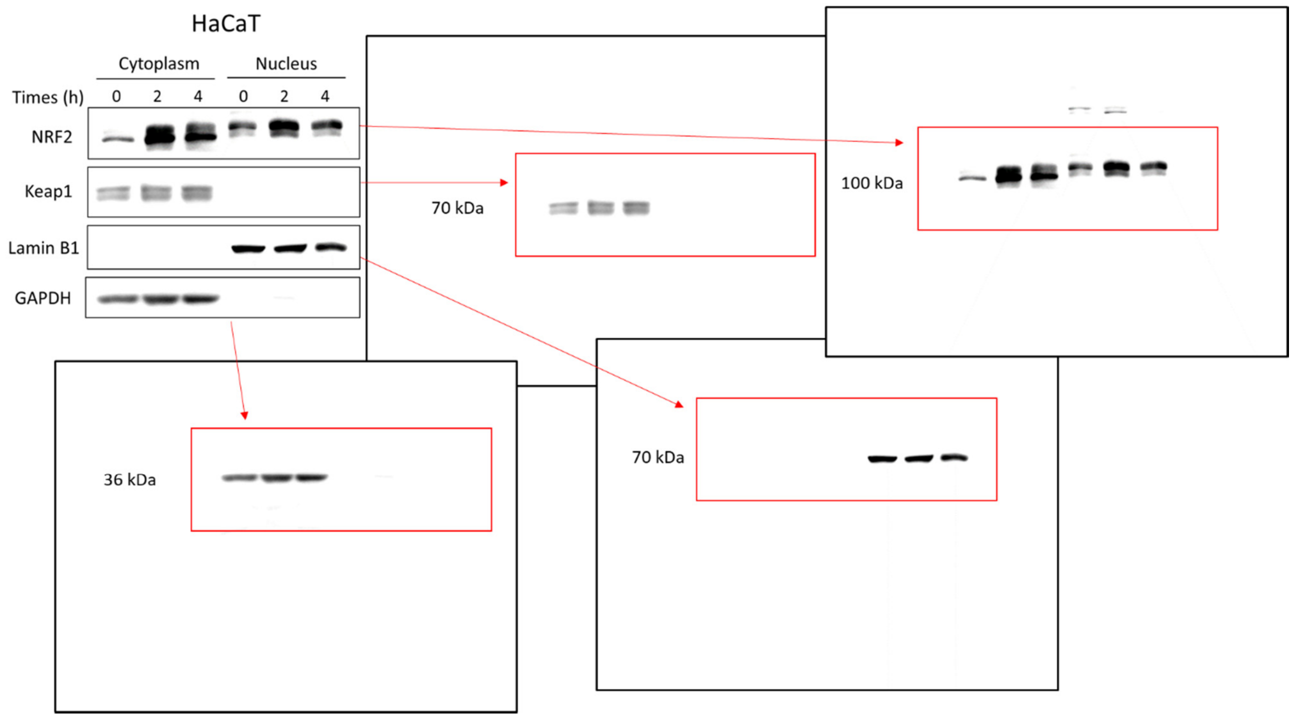

Supplementary Figure S7. Raw blot data related to Figure 4A,B,E.
